# Pictures Library of Smoking Cravings: Development and Verification of Smokers and Non-smokers

**DOI:** 10.3389/fpsyt.2021.719782

**Published:** 2021-08-16

**Authors:** Zhongke Gu, Hui Zheng, Zhifei Yin, Huiting Cai, Yongqiang Li, Chunchun Zhao, Yujia Zhai, Kai Xu, Lian Xue, Xingjun Xu, Ying Shen, Ti-Fei Yuan

**Affiliations:** ^1^Department of Sport and Health Sciences, Nanjing Sport Institute, Nanjing, China; ^2^Shanghai Key Laboratory of Psychotic Disorders, Shanghai Mental Health Center, Shanghai Jiao Tong University School of Medicine, Shanghai, China; ^3^Rehabilitation Medicine Center, The First Affiliated Hospital of Nanjing Medical University, Nanjing, China; ^4^Department of Psychology, Zhejiang Normal University, Jinhua, China; ^5^Scientific Experiment Center, Nanjing Sport Institute, Nanjing, China; ^6^Co-innovation Center of Neuroregeneration, Nantong University, Nantong, China; ^7^Translational Research Institute of Brain and Brain-Like Intelligence, Shanghai Fourth People's Hospital Affiliated to Tongji University School of Medicine, Shanghai, China

**Keywords:** cue-induced craving, picture database, expectation model, cigarette picture, smoking pictures

## Abstract

**Background:** The cue-induced craving by addiction related materials is commonly employed in addiction research; however, no existing standardized picture database based on the expectation model of craving has been developed. We prepared and validated a Pictures Library of Smoking Cravings (PLSC) in this study.

**Methods:** We captured pictures 366 smoking and 406 control pictures (matched in content). We selected 109 smoking pictures and 115 control pictures and asked participants to provide ratings of craving, familiarity, valence, and arousal induced in them. Participants were divided into three groups: non-smokers (*n* = 211), light smokers (*n* = 504), and heavy smokers (*n* = 101).

**Results:** The results showed that smoking pictures evoked a greater craving, familiarity, and arousal than control pictures in smokers (*p*s < 0.01). In addition, craving caused by smoking pictures was positively associated with the Fagerström test for nicotine dependence score in dependent smokers.

**Conclusions:** Overall, the contemporary results showed that PLSC is effective and can be used in smoking-related studies.

## Introduction

Smoking cue reactivity craving is a robust neuro biomark for nicotion addiction (1). Craving is an essential defining hallmark of addiction (2) and can be employed to explain the initiation, maintenance, and relapse of compulsive addiction behavior (3, 4). The expectation model defines substance-related craving as a subjective state motivated by the incentive properties of positive outcome expectancies in addiction behavior (5), which can also be used to explain human desires (6). In the cognitive model of addiction divides craving into automatic and non-automatic processes in frontostriatal circuits (7–9). However, despite the large number of studies using smoking-related pictures as an automatic cue in the cue-induced craving paradigm (10), only a few standardized picture libraries have been developed to study smoking-related non-automatic processes (11).

The lack of structured picture libraries hinders the study of non-automatic processes of craving. Only three smoking-related picture libraries have been previously published. The first is the most widely distributed image series called the international smoking image series (with neutral counterparts) (12). In this image series, 300 subjects (70 smokers, 145 men) were included to rate 121 smoking-related pictures and 69 neutral pictures in terms of interest, valence, arousal, and smoking tendency. The second is the International Affective Picture System (IAPS), which only includes a few smoking-related pictures (13). The latest library is the Geneva Smoking Pictures, in which 92 (46 dependent and 46 non-dependent) subjects were recruited to rate 60 pictures into three types: 1. the product itself (such as cigarette packs, lit cigarettes), 2. smoking behavior (such as smoking a cigarette, rolling a cigarette), and 3. tobacco-related cues by valence, arousal, and dominance (14). The above studies recruited only a few subjects (<300) and rated a cluster of mixed pictures on the emotional dimensions (valence, arousal), with an uncontrolled background of the picture. In particular, none of the picture libraries considered the smoking process (e.g., just lit a cigarette, inhaled the first puff) with content-matched pictures.

Considering the above circumstances, it is vital to develop a standardized and structured smoking pictures library: Pictures Library of Smoking Cravings (PLSC). As such, we aim to analyze the IAPS test method (13), the familiarity of the constructed pictures, the craving inducing effect, and the relationship between craving and the severity of nicotine dependence. Future studies can flexibly match the required experimental materials depending on their requirements. The rates of these datasets are presented on the open science framework (OSF) website (https://osf.io/pb58y/). To provide effective tools for further refinement of the neural mechanisms involved in smoking craving (15). We aim to developed a picture library of the smoking process from three aspects: 1. divide the non-automated smoking process into nine stages (described in the Methods section) to capture the dynamic changes of smoking-related craving, 2. to create the natural control pictures, use objects similar to those used in cigarette products and make gestures (should be natural, such as brushing teeth, pencils) similar to those used in smoking, and (3) unify the aforementioned film standard with given corresponding standardized methods, including detailed control of shooting distance, model positions, lighting, focal length, and background processing (16).

We hypothesized that 1. compared with control pictures, smoking pictures can induce higher cravings among smokers, whereas among non-smokers, both types of pictures cannot induce cravings, and 2. the craving induced by our pictures can predict the severity of nicotine dependence.

## Materials and Methods

### Participants

Smoking participants were enrolled from website (age ≥ 18 years old, *N* = 977), and they completed the online version of 224 PLSC. Participants were invited to assess their subjective experience of craving based on a 11-point Likert scale from 0 (not at all) to 10 (very much). Participants were habitual smokers and had no other mental illnesses. This study was based on the planned missing data design, and each picture was rated more than 120 times (17). A shorter response time, lower even-odd consistency measure, and lengthy strings of invariant responses are indicative of low-quality data (18). The requirements of our dataset included no more than one-third of consecutive options, no contradictions in information (for example, the date of birth should correspond to the self-reported age), and a final signature is provided to show agreement to voluntarily take a serious answer. After this quality check, 816 subjects (male 616, female 200) finally met the above requirements: the mean age was 28.98 (SD = 10.30), the mean number of years of education was 13.55 (SD = 2.42), and the baseline craving was 25.69 (SD = 24.74). Using the Fagerström test of nicotine dependence (FTND), we divided the subjects into non-smokers (*n* = 211), light smokers (FTND < 5, *N* = 504), and heavy smokers (FTND ≥ 5, *N* = 101, as nicotine dependent) (more details are shown in Table 1).

**Table 1:** Demographic description of PLSC.

	**Mean** **±** **SD/Ratio**				***F/x*** **^2^ value**	***df***	***p-*** **value**	***Partial η*** **^2^**
	**HS** (***n*** **= 101)**	**LS** (***n*** **= 504)**	**Smoker** (***n*** **= 605)**	**NS** (***n*** **= 211)**				
Age	35.68 ± 10.87	28.88 ± 10.02	30.00 ± 10.5	26.00 ± 9.19	32.63	2,813	**<0.0001**	0.0731
Gender (male %)	78 (77.23%)	409 (81.15%)	487 (80.50%)	129 (61.14%)	32.39	2	**<0.0001**	/
Education year	12.99 ± 3.18	13.56 ± 2.33	13.5 ± 2.50	13.79 ± 2.19	3.77	2,813	**0.0235**	0.0092
FTND	6.12 ± 1.34	1.47 ± 1.35	2.25 ± 2.20	1.36 ± 1.51	500.52	2,813	**<0.0001**	0.5519
Baseline Craving	49.39 ± 25.87	29.63 ± 21.98	32.90 ± 23.80	4.91 ± 12.58	184.45	2,813	**<0.0001**	0.3121

*HS, heavy smoking participants; LS, light smoking participants; NS, no smoking participants; FTND, Fagerström test of nicotine dependence; F-test was performed among HS, LS, and NS groups. There is significant difference between smoker and NS in age, gender, FTND, baseline craving (ps < 0.001)*.

### Photography Information

**Figure 1:**
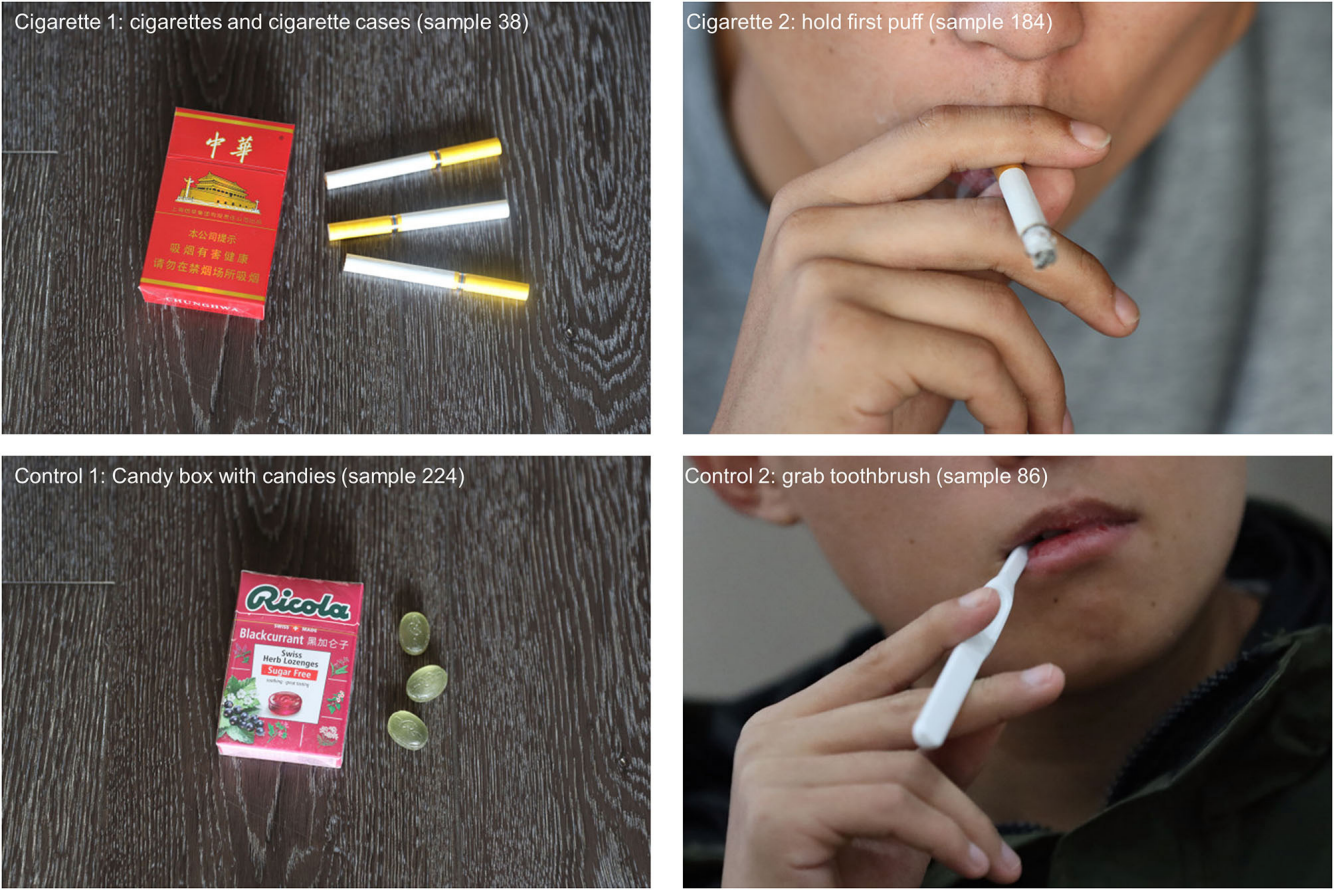
Four sample pictures from the PLSC (Pictures Library of Smoking Cravings). The cigarette related picture No. 38, No. 184, and natural context control picture No. 224, No. 86. The natural context control pictures have similar picture structure.

The pictures were captured using a high-resolution digital single-lens reflex camera mounted on a tripod. The focal length used was 24–70 and 70–200 mm. To make each scene as attractive as possible, the shutter speed and aperture were automatically adjusted, the maximum aperture of both lenses was f/2.8, and autofocus was used. All pictures were captured under natural ambient light and indoor ordinary lighting (indoor LED light source, color temperature 4500 K) without supplementary light. The camera and lens are in horizontal state during shooting, the distance from the model to the tripod center was 150 cm (24–70 mm)/300 cm (70–200 mm), and the height of the center of the camera on the tripod was 120–140 cm, which is flush with the human line of sight. In addition, the background was blurred; thus, the pictures taken were as standard as possible while remaining natural. Pictures were post-processed using Adobe Photoshop CC 2019 and were exported in the JPEG format (2,700 × 1,800 pixels). The luminance was manipulated using SHINE color (19). The pictures have been uploaded to the PLSC website, which is free and open to researchers. In terms of standardization, we chose objects shaped like cigarettes/cigarette boxes, which are visible in daily life (pencil, toothbrush, and sugar box), and asked the model to hold these objects with gestures similar to that of holding cigarettes for control pictures (Figure 1).

**Figure 2:**
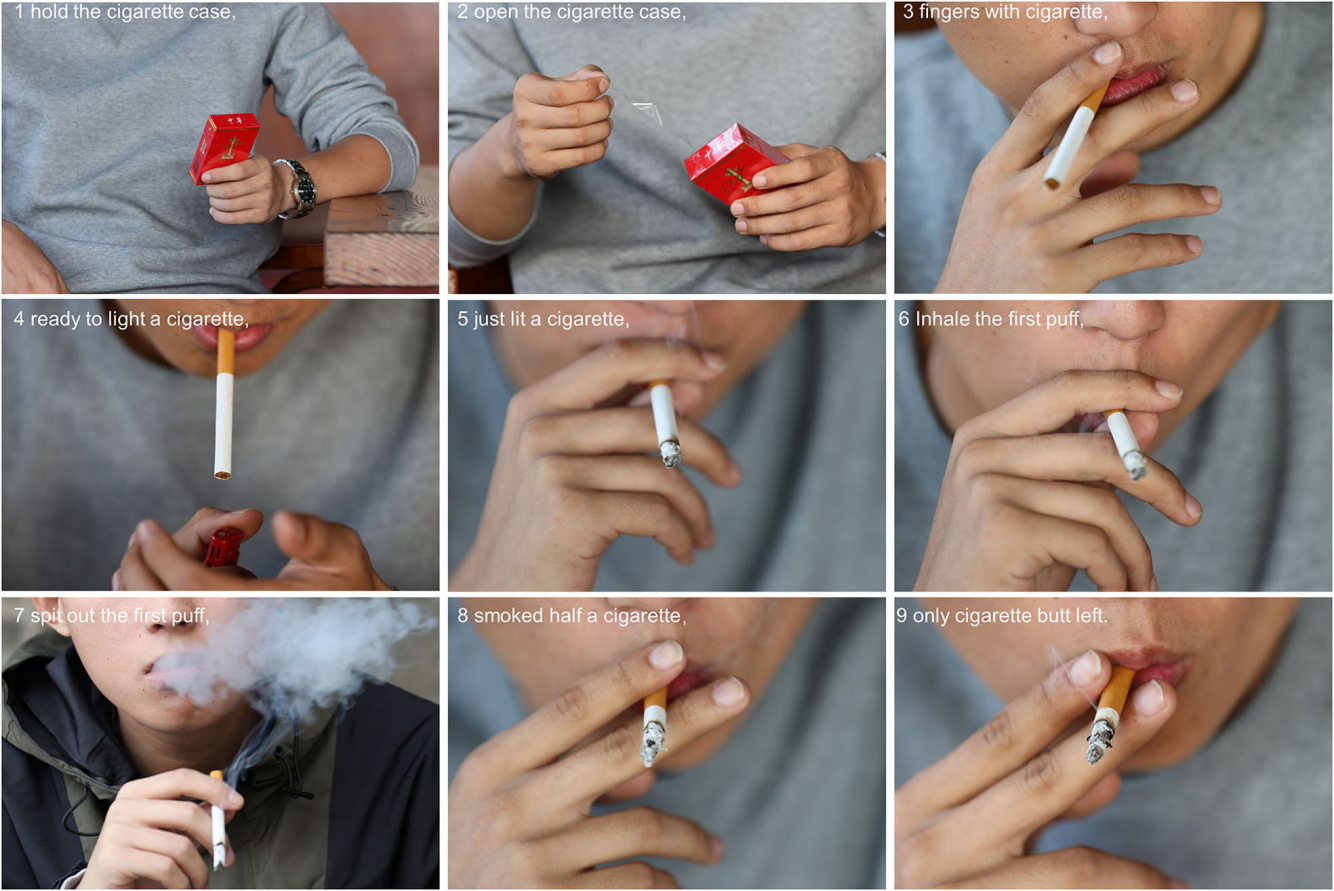
The nine stages of smoking behavior. Participants who smoke a cigarette will follow these stages, and we assume that during this process, the level of craving will change.

To represent smoking behavior, we divided smoking behavior into nine stages: 1. hold a cigarette case, 2. open a cigarette case, 3. ready to light a cigarette, 4. fingers holding a cigarette, 5. just lit a cigarette, 6. inhale the first puff, 7. spat out the first puff, 8. smoked half a cigarette, and 9. only cigarette butts left (Figure 2). Our database included 109 smoking pictures, with 12 pictures for each stage [two (gender, male and female) × three scenes (middle, close, and close-up) × two (with no human face)], and one picture of three standing cigarettes and a cigarette box in an overhead shot as a benchmark. Our extended image library (366 smoking and 406 control pictures) included three perspectives: left, center, and right, and the benchmark was extended to 1–5 cigarettes.

### Measures

First, participants were asked to complete the FTND (20). Then, the participants evaluated each picture separately, and each picture was shown once (www.wjx.cn). For each image, the participants were required to rate their craving [from 0 (“not at all”) to 10 (“very”)], valence [from 0 (“negative”) to 1 (“positive”)], arousal [from 0 (“not at all”) to 10 (“very much”)], familiarity [from 0 (“not at all”) to 10 (“very much”)], and related to smoking [0 (“no”), 1 (“yes”)]. In total, each participant had to complete 36 trials (half smoking picture, half control). The entire dataset was divided into 18 sub-datasets (average completion time = 656.44 s). In addition, each participant had to provide the FTND score, the baseline craving scale, and basic demographic information (sex, age, and number of years of education).

### Statistical Analysis

All statistical tests were performed using jamovi (www.jamovi.org/). Continuous variables (such as the Likert scale with normal distribution) were expressed as mean ± standard deviation (SD), in which logarithmic model parameters were logarithmically transformed; non-normal variables are reported as the median (interquartile range). Continuous normally distributed variables were compared using the variance test. *p* < 0.05 was considered significant. To control multiple comparison deviations, Bonferroni correction was used. The Pearson correlation and linear regression was used to test the correlation between ratings for cigarette-related pictures, and the correlation between pictures that evoked craving and the FTND score. The rate score is the predicted variable and the FTND score is the predict variable.

## Results

**Figure 3:**
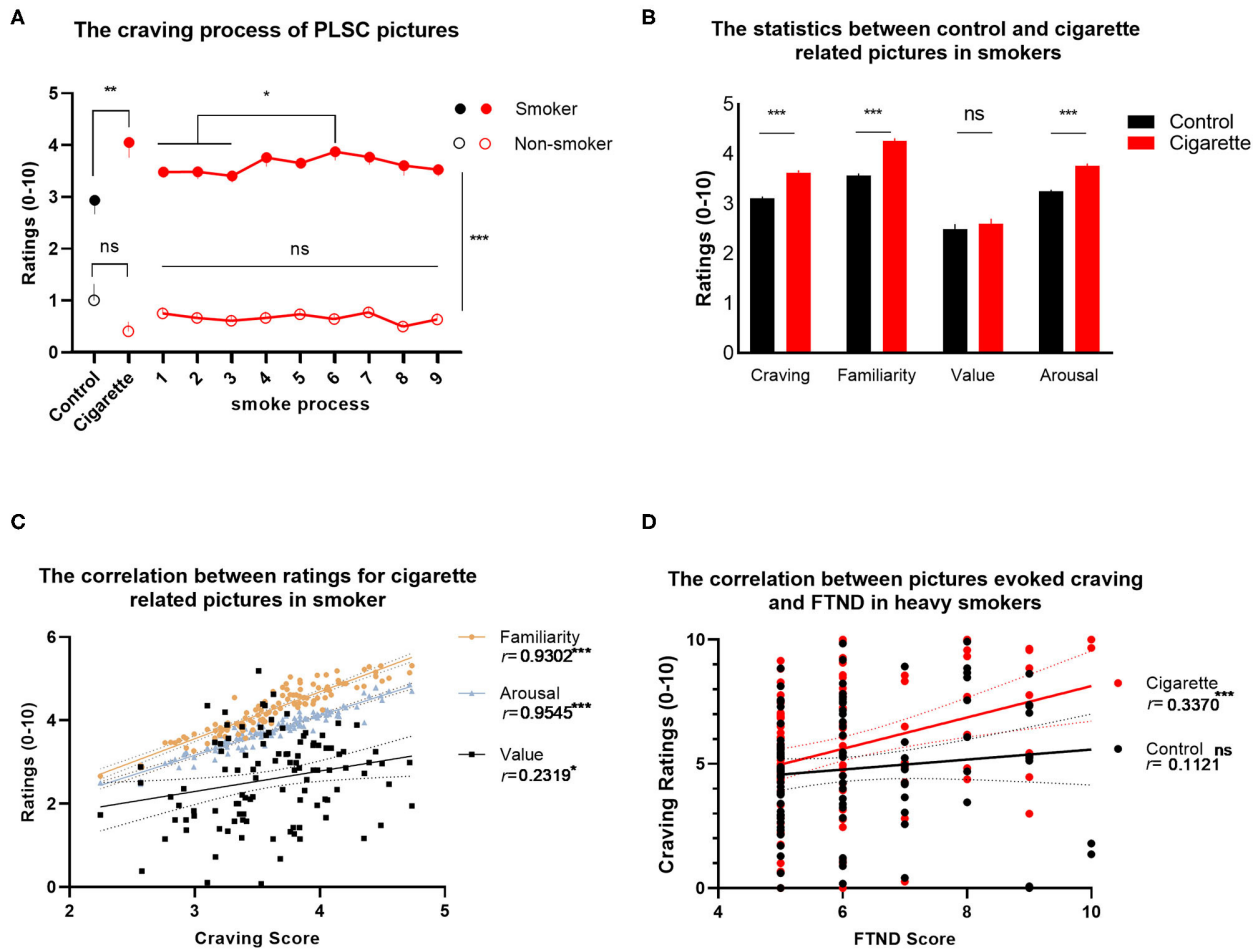
Statistical analysis of PLSC data. **(A)** The craving process of PLSC pictures. For smokers, the craving for smoking related pictures is higher than that for natural content control pictures. The sixth stage of smoking, namely, inhale the first puff, showed stronger induced craving than the preparation stage (1–3). The level of picture induced craving in smokers was higher than that in non-smokers. **(B)** The statistical analysis between control and cigarette-related pictures in smokers. The cigarette related picture had shown significantly higher values of craving [*t*(222) = 8.55, *p* < 0.001], familiarity [*t*(222) = 9.81, *p* < 0.001], and arousal [*t*(222) = 8.47, *p* < 0.001 **(B)**] than control pictures. **(C)** The correlation between ratings for cigarette-related pictures. There are significant positive correlation between craving and familiarity [*r*(109) = 0.93, *p* < 0.001]; between craving and valence [*r*(108) = 0.23, *p* = 0.016]; and between craving and arousal [*r*(109) = 0.95, *p* < 0.001]. **(D)** The correlation between pictures that evoked craving and the FTND score. The cigarette related picture induced craving is significant correlated with the FTND score in heavy smokers. The craving score of cigarette related pictures explained a significant amount of the variance in the FTND score [*F*_(1, 102)_ = 13.07, *p* = 0.0005, *r*^2^ = 0.1136]. ns, no statically significant, ^*^p < 0.05, ^**^p < 0.01, ^***^p < 0.001.

To test if addiction pictures could induce higher cravings among smokers, we used the independent *t*-test. The results showed that smoking pictures (*M* = 4.05, SD = 3.03) evoked higher craving than control pictures [*M* = 2.94, SD = 2.77, *t*(213) = 2.814, *p* = 0.0054] in smokers (Figure 3A). The following *post-hoc* analysis showed that process 6 (inhale the first puff) induced greater cravings than processes 1, 2, and 3 in smokers (*ps* < 0.05). However, in non-smokers, no significant difference between each kind of picture was observed. Dynamic changes in cravings were observed during the sequence of smoking, which could be induced by pictures of different smoking states.

We used the independent *t*-test to verify the stability of emotional dimensions for PLSC in smokers. The results showed that smoking pictures demonstrated significantly higher values of craving [*t*(222) = 8.55, *p* < 0.001], familiarity [*t*(222) = 9.81, *p* < 0.001], and arousal [*t*(222) = 8.47, *p* < 0.001, Figure 3B] than control pictures. For smoking pictures, the Pearson correlation analysis showed a strong correlation between craving and familiarity [*r*(109) = 0.93, *p* < 0.001]; between craving and valence [*r*(108) = 0.23, *p* = 0.016]; and between craving and arousal [*r*(109) = 0.95, *p* < 0.001, Figure 3C].

In heavy smokers, a simple regression was used to predict craving caused by smoking pictures from the severity of nicotine dependence. The craving score of cigarette related pictures explained a significant amount of the variance in the FTND score [*F*_(1, 102)_ = 13.07, *p* = 0.0005, *r*^2^ = 0.1136]. The regression coefficient (*B* = 0.632) indicated that an increase of 1 unit in the FTND score, on average, led to a discrete 0.632 craving score for smoking pictures (Figure 3D). This may suggest that the picture library has a strong ability to identify patients with nicotine dependence.

To control potential influencing factors, an independent sample *t*-test was used to detect whether the presence or absence of a human face, shooting angle, model's sex, model's actions, and the scene would interfere with the craving state of participants. There was no significant difference among any ratings (*ps* > 0.05, craving, familiarity, potency, and arousal). The above results indicate the success of our developed picture library (for detailed scoring results of the images in each chapter, please see the Author's Note).

## Discussion

We attempted to establish a novel, standardized, and structured library of smoking pictures. We developed a library of 109 smoking-related pictures and 115 control pictures matched in content based on the smoking process. The pictures were rated for subjective craving, valence, and arousal according to the emotional validation procedure developed by the IAPS (13). We observed that the use of static pictures can also induce a dynamic craving process; inhaling the first puff of a cigarette induced the highest craving among all the nine stages of the smoking process. In addition, the ranking of the static cigarette pictures (only a cigarette and cigarette box) was still higher than that of pictures such as just lit a cigarette and spat out the first puff (22/109 of all cigarette pictures). There has been no dynamic elasticity study of nicotine dependence. A previous study of alcohol use disorder showed that craving intensity increased throughout the day (pre-drinking), followed by alcohol consumption, which significantly reduced craving intensity (21).

Our results support our hypotheses and are congruent with the expectation model of craving. For non-smokers, no significant difference was observed between our smoking pictures and control pictures. For smokers, the smoking pictures induced more inner automic cravings of participants than control pictures (22). This may coincide with the abnormal neural mechanism of the frontal -striatum circuit in smokers (23, 24). We found that smokers still rated higher cravings for control pictures with common placement like smoking pictures. This may be because craving was induced during the rating process, and a habituation reaction was formed, leading to an increase in craving for neutral materials (25). For heavy smokers, smoking pictures in the PLSC induced a higher craving, and the more severe the nicotine dependence, the higher the craving score it induced. This may be due to the abnormal reward processing of dependent smokers, resulting in an automated increase in the subjective value of smoking stimulation (26).

Finally, when analyzing the concept of craving, we should consider the entire process, that is, craving includes two stages—approach and avoidance—and the contradictions that exist between the approach and avoidance (27). When we evaluated the valence of pictures, we found that there was no significant difference between the valence of smoking-related pictures and that of the control pictures, which may be caused by the motivation to avoid smoking (28).

This study has two limitations: first, we did not control the relationship between the last smoking time and rating time, although some studies have claimed that the last cigarette is not significantly related to craving (29). This sample has a loose population distribution, and its coverage is insufficient. There are differences in age, gender, and educational levels among the three groups. But if you need to cover such a complex group, you need a huge amount of data. We may need to use the planned missing data design to solve this issue (17). Third, the familiarity of some control pictures was considerably different, so researchers who want use this picture dataset need matching materials before the experiment. Finally, this material is mainly used in Asian research, such as behavioral experiments (point detection, GoNogo, Stroop, etc.), neuroimaging (event-related ERP, task status MRI, task MRI, task NIR, etc.).

## Data Availability Statement

The datasets presented in this study can be found in online repositories. The names of the repository/repositories and accession number(s) can be found below: https://osf.io/pb58y/.

## Ethics Statement

The studies involving human participants were reviewed and approved by the Ethics Committee of the First Affiliated Hospital of Nanjing Medical University. The patients/participants provided their written informed consent to participate in this study.

## Author's Note

The pictures have been uploaded to the osf website (https://osf.io/pb58y/), which is free and open to researchers.

## Author Contributions

YS and T-FY had full access to all the data in the study and take responsibility for the integrity of the data and the accuracy of the data analysis. HZ, YS, and T-FY designed the trial. ZG and HZ wrote the original draft as well as review and editing of the current manuscript. CZ, YZ, YL, and KX performed the data analysis. LX, ZY, YZ, and XX performed the study procedures. HC, YS, and T-FY provided critical revisions to the manuscript. YS was responsible for funding acquisition. All authors have reviewed and approved the final version of the manuscript for submission.

## Conflict of Interest

The authors declare that the research was conducted in the absence of any commercial or financial relationships that could be construed as a potential conflict of interest.

## Publisher's Note

All claims expressed in this article are solely those of the authors and do not necessarily represent those of their affiliated organizations, or those of the publisher, the editors and the reviewers. Any product that may be evaluated in this article, or claim that may be made by its manufacturer, is not guaranteed or endorsed by the publisher.
